# A brief gestalt intervention changes ultrasound measures of tongue movement during breastfeeding: case series

**DOI:** 10.1186/s12884-021-04363-7

**Published:** 2022-02-01

**Authors:** Pamela Sylvia Douglas, Sharon Lisa Perrella, Donna Tracy Geddes

**Affiliations:** 1 Possums & Co., PO Box 5139, West End, Brisbane 4101 Australia; 2 School of Nursing and Midwifery, Griffith University, Nathan, Australia; 3 Primary Care Clinical Unit, The University of Queensland, Herston Road, Herston, Australia; 4 Geddes Hartmann Human Lactation Research Group, The University of Western Australia, Perth, Australia

**Keywords:** Breastfeeding, Latch, Ultrasound, Infant suck, Nipple pain, Lactation

## Abstract

**Background:**

Lactation consultants frequently advise adjustments to fit and hold (or positioning and attachment) with the aim of optimising intra-oral nipple placement. However, approaches to fit and hold vary widely, with limited evidence of benefits, and effects of fit and hold on infant tongue movement have not been examined. The aim of this preliminary study was to investigate whether a gestalt breastfeeding intervention alters tongue movement, using measurements from ultrasound imaging to determine nipple placement and intra-oral nipple and breast tissue dimensions.

**Methods:**

Ultrasound measurements were conducted in five breastfeeding dyads, infants aged 4–20 weeks, while feeding in their usual or ‘standard’ position and again after brief application of gestalt principles of fit and hold. Four of the mother-baby pairs, who had received comprehensive lactation support, reported persisting nipple pain. Three of these infants had difficulty latching and fussed at the breast; three had been diagnosed with oral ties. A fifth pair was breastfeeding successfully.

**Results:**

Ultrasound demonstrated that the distance from nipple tip to junction of the hard and soft palate decreased, intra-oral nipple and breast tissue dimensions increased, and nipple slide decreased after a brief gestalt intervention.

**Conclusion:**

These preliminary findings suggest that changes in fit and hold impact on infant tongue movement and contour. Further research investigating short- and long-term outcomes of a gestalt breastfeeding intervention in larger cohorts is required.

**Supplementary Information:**

The online version contains supplementary material available at 10.1186/s12884-021-04363-7.

## Background

At the time of giving birth, most women want to breastfeed. However, parents report commencing formula because of maternal breast and nipple pain, unsettled infant behaviour, infant weight gain concerns, or perceptions of low supply (the latter often because of unsettled infant behaviour) [1–4]. Up to 79% of breastfeeding women experience nipple pain in the first eight weeks post-birth [5]. These problems increase risk of postnatal depression [6, 7], yet women with breastfeeding difficulties receive a great deal of conflicting advice [8, 9].

The physiologic approach to breastfeeding initiation, including skin-to-skin contact postpartum, has been a major advance in the field of clinical breastfeeding support over the past two decades, with positive impacts on breastfeeding outcomes [10–13]. But the range of fit and hold (positioning and attachment) interventions currently applied by breastfeeding support professionals, including ‘baby-led’ or mammalian methods, have not been demonstrated to help treat pain or unsettled infant behaviour after discharge from the maternity hospital, including in randomised controlled trials [14–21].

For example, one popularly applied fit and hold technique teaches women to shape their breast and apply a cross-cradle hold as they bring the infant on. In 2002 this technique, when taught to hospital midwives in Bristol, UK, was shown in a prospective cohort study of 1171 new mothers to increase the rate of breastfeeding at six weeks post-birth relative to usual care [22]. But in a 2016 Australian retrospective study of the medical records of 653 pairs, this same technique was also shown to be associated with an increased incidence of nipple trauma, attributed to nipple malalignment and facio-mandibular asymmetry [23].

A 2020 meta-analysis which searched the literature for the benefits of ‘laid-back breastfeeding’ or ‘biological nurturing’ selected 12 studies, 11 randomised controlled trials (RCTs) and 1 quasi-randomised controlled trial. This meta-analysis found that when the biological nurturing approach is applied in the first three days post-birth, at follow-up after a period of between 3 days to 8 weeks there was a significant reduction in nipple pain and nipple trauma, though no difference in position comfort [24]. 11 of these studies were published in Chinese. The Italian RCT, published in English, compared 90 breastfeeding women who applied biological nurturing from birth with 98 who received usual care. The biological nurturing group showed a decreased incidence of sore nipples, cracked nipples, engorgement and mastitis at the time of hospital discharge, and this finding was confirmed 7 days post-discharge. Yet the incidence of these problems increased slightly in the biological nurturing group compared to usual care at 30 days post-discharge, with no change in breastfeeding rates either at discharge or up to 4 months post-birth [25]. A 2021 Chinese RCT of 504 pairs demonstrated that implementing baby-led self-attachment from birth resulted in a 12% increase in exclusive breastfeeding at day 3, and an 8 and 5% decrease in the number who reported nipple pain at 3 days and 3 months postpartum, respectively [26].

Although these studies demonstrate modest preventive benefits of biological nurturing when applied from the first days of life, a 2013 Swedish randomised controlled trial of 103 of mothers with babies up to 16 weeks of age with severe latch-on difficulties found that a ‘baby-led’ or skin-to-skin intervention did not make it more likely that the infant would latch-on [27].

Infants who show signs of inability to latch, back-arching, fussing, and pulling off the breast are at risk of pharmaceutical interventions for inappropriate diagnoses of gastro-oesophageal reflux or maternal elimination diets for inappropriate diagnoses of allergy. These treatments have been demonstrated to result in unintended outcomes, including increased risk of true allergy [28–35]. Similarly, breastfed babies demonstrating back-arching, fussing, and pulling off the breast or whose mothers experience nipple pain are currently at risk of unnecessary lingual and/or labial frenotomy and courses of bodywork therapy, which may also result in unintended outcomes [36–44]. Given these concerns, it is essential that every effort is made to optimise breastfeeding when problems emerge, prior to considering medical or surgical intervention or bodywork treatments. Yet current fit and hold approaches, including ‘laid-back breastfeeding’, have not been demonstrated to be effective therapeutic interventions for babies who present with breastfeeding problems in the community.

### Ultrasound and vacuum studies elucidate the biomechanics of the infant suck cycle in breastfeeding

Ultrasound imaging demonstrates that when the infant’s tongue is up during breastfeeding, the mid-tongue rests against the hard palate, the junction of the hard and soft palate, and the soft palate, sealing the oral cavity from the pharynx. An oral cavity seal is also required at the breast-face interface to generate baseline vacuum. When the tongue is up, the intra-oral depth (IOD) is measured between the junction of the hard and soft palate (HSPJ) and the highest part of the tongue. The IOD when the tongue is up is often but not always 0 mm in successfully breastfeeding pairs. That is, the apposition of the tongue and palate which comprises the pharyngeal seal mostly occurs between the mid-tongue and the junction of the hard and soft palate, but in 10% of cases may occur more posteriorly, between the soft palate and the most posterior part of the tongue, also known as the tongue base (which is not visible on oral examination) [45–47]. The base of the tongue has no anatomic connection with the lingual frenulum [41].

The distance between junction of the hard and soft palate and the nipple tip is referred to by the acronym NHSPJD. When reflex depression of the mandible commences, the anterior and mid-tongue depress as a single unit, moving *en bloc* and in tandem with the mandible. The soft palate tracks the base of the tongue, which also moves inferiorly with the mid-tongue, both mid-tongue and base of the tongue tracking the mandible. Intra-oral vacuum (not tongue movement) drives milk transfer during breastfeeding, in tandem with contraction of the alveolar glands and dilation of the ducts during milk ejection. Peak vacuum is achieved when the mandible is extended, and is typically twice that of baseline vacuum [48]. The nipple ducts may become visible as milk fills the intra-oral space. The intra-oral space is bordered distally by the nipple tip, proximally by the soft palate in apposition with the tongue base, superiorly by the hard palate, and inferiorly by the dorsal surface of the tongue, and does not contain air [45–47, 49].

When the mandible is fully depressed and the tongue is down, the intra-oral depth (IOD) or depth axis continues to be measured as a line drawn between the junction of the hard and soft palate and the highest part of the tongue. When the mandible is maximally depressed, the NHSPJD continues to be measured between the nipple tip and the depth axis. The difference in NHSPJD between tongue up and tongue down indicates the degree of horizontal movement or slide of the intra-oral nipple and breast tissue during sucking [45, 47]. In validation studies, nipple and breast tissue width dimensions (referred to in previous ultrasound studies as ‘nipple compression’ or ‘nipple diameter’) are measured at a distance of 2, 5, 10, and 15 mm proximal to the nipple tip in tongue up and tongue down [45]. The anterior portion of the tongue begins to rise slightly before the mid-tongue reaches its most inferior point. As the mid-tongue lifts to the palate with the rise of the mandible, milk passes between the soft palate and the tongue base [45–47].

The following ultrasound measures are associated with less maternal nipple pain and improved milk transfer:

- Decreased distance between the nipple tip and junction of the hard and soft palate (NHPSPJ), both when the mandible is up and in full mandibular depression (‘improved’ nipple placement);
- Expanded nipple and breast tissue dimensions;
- Increased intra-oral dimensions (IOD, or ‘improved’ tongue shape);
- Decreased nipple slide (distance the nipple tip moves between tongue up (TU) and tongue down (TD)) [45–48, 50].

### The gestalt method is a novel clinical interpretation of the findings of two-dimensional ultrasound studies and vacuum studies, corroborated by real-time MRI

The tongue is a muscular hydrostat which changes shape without changing volume [51]. In the gestalt (pronounced 'ger-shtolt') biomechanical model of infant suck and swallow, the tongue is conceptualised as a supple, adaptive organ which dynamically responds to and moulds around available intra-oral nipple and breast tissue, rather than as a forcible driver of nipple compression and nipple shape [35].

The two-dimensional ultrasound measures of the suck cycle are interpreted in the gestalt model as markers of three-dimensional increases in intra-oral breast tissue volume, to which the tongue conforms by changing shape. That is, measured changes of tongue surface or nipple placement relative to other intra-oral anatomic structures and of nipple and breast tissue dimensions, whilst previously interpreted as measures of tongue movement or mobility, are conceptualised in the gestalt biomechanical model as proxy measures of intra-oral breast tissue volume, to which the dorsum of the tongue moulds [35, 52].

The gestalt biomechanical model of infant suck and swallow proposes that suboptimal fit between infants’ and their mothers’ diverse anatomies may create a vector of force in the infant’s mouth which conflicts with the directions of vacuum generated during mandibular depression, in the context of the seal formed by the face-breast bury and apposition between the tongue and hard or soft palate. This conflicting vector of force, referred to clinically as ‘breast tissue drag’, results in nipple pain and/or fussy infant behaviour at the breast [35, 52].

In the gestalt model, elimination of conflicting vectors of force intra-orally (that is, elimination of breast tissue drag) allows peak vacuum to achieve optimal intra-oral breast tissue volume. The impact of peak vacuum is diffused over the largest possible surface area of the intra-oral nipple-areolar complex and breast. This diffusion of tensile pressure is hypothesised to prevent excessive stretching. That is, it prevents the epithelial damage which may result when a high tensile load is focussed upon a small surface area. Because most alveolar glandular tissue is within a three-centimetre radius of the nipple, optimal intra-oral breast tissue volume optimises milk transfer, satiety, and weight gain [35, 52–54].

The gestalt biomechanical model has been corroborated in 2020 by findings of a real-time MRI series of 12 successfully breastfeeding mother-baby pairs [55]. Real-time MRI confirms that the anterior and mid-tongue track the mandible *en bloc*; that there is no air in the intra-oral space during sucking and swallowing; that upper lip position is usually neutral during suckling; and that the tongue tip rests on the lower gum without protruding beyond the lips during suckling. Mills et al. demonstrate that the infant’s soft palate remains in dynamic apposition with the tongue base, as the latter moves anteriorly and posteriorly. In the swallow phase, the soft palate elevates, allowing the bolus of milk to pass under it.

In this paper, the terms ‘mandible up’ and ‘maximum mandibular depression’ are used synonymously with tongue up and tongue down, respectively, to emphasise that the infant suck cycle is not driven by independent tongue up and tongue down movements, but that the anterior and mid-tongue move *en bloc,* in tandem with and tracking the mandible [47, 49, 55]. Also, in this paper, the term ‘nipple and breast tissue dimension’ refers to what has previously been characterised as ‘nipple compression’ or ‘nipple diameter’, in order to acknowledge that, depending on nipple length and width, intra-oral breast tissue typically includes subareolar glandular tissue, and perhaps even more proximal glandular tissue, in addition to nipple [56]. The synonymous terms nipple and breast tissue dimensions, width, or diameter also acknowledge that what has been previously interpreted as compression by the tongue is a change of shape resulting from tensile forces acting on elastic tissue [35].

In the gestalt model, patterns of infant back-arching, fussing, and pulling off the breast during breastfeeding are understood to commonly result from positional instability, which generates breast tissue drag. The term ‘positional instability’ describes a position in which the infant signals either subtle or significant discomfort, that is, experiences challenges with motoric postural control. A baby who is positionally stable may nevertheless be subject to breast tissue drag, resulting in maternal nipple pain. Therefore, a gestalt clinical intervention aims to stabilise the fit between the infant and his or her mother’s body and breast, which eliminates breast tissue drag. The gestalt method builds on the foundations of ‘laid-back breastfeeding’ positioning [24], but integrates a range of other strategies to optimise suckling biomechanics (See ‘Key elements of the gestalt approach to clinical breastfeeding support’, Additional file 1). This approach has implications for multiple aspects of usual lactation support, including for minimising unnecessary pharmaceutical, surgical and bodywork interventions [35].

### Aim

Our study investigates the hypothesis that a gestalt intervention results in changes in nipple placement, infant tongue position and shape, and nipple and breast tissue dimensions during breastfeeding.

## Methods

### Design

PD designed the study, in which breastfeeding pairs consented to a brief gestalt intervention in the laboratory. The methods were designed in accordance with usual guidelines, which recommend a fit and hold intervention for problems of nipple pain and fussiness at the breast. The brief intervention focussed on communicating key strategies of a gestalt intervention, in a 5–10 min time interval. (See ‘Key elements of the gestalt approach to clinical breastfeeding support’, Additional file 1.) Ultrasound measurements to determine the effects of the intervention were taken immediately prior to and immediately after the intervention. The study was approved by the Human Ethics Committee of The University of Western Australia. Although there was no formal Patient and Public Involvement in the design, PD is a general practitioner who specialises in breastfeeding medicine. She is an International Board Certified Lactation Consultant (IBCLC), who has worked with breastfeeding mothers for 30 years. Her experience of the needs communicated by breastfeeding women over this time has informed both the design of this preliminary evaluation and the previous development of the gestalt intervention [35, 52].

### Setting

The study was conducted in the Geddes Hartmann Human Lactation Research Group laboratory located in the King Edward Memorial Hospital, Perth, Western Australia.

### Sample

In 2016, an International Board Certified Lactation Consultant (IBCLC), who is also a qualified midwife and post-doctoral research fellow, recruited five breastfeeding dyads in Perth, Australia. Informed signed consent was obtained from the mothers for themselves and their infants to participate in ultrasound analysis before and after a gestalt intervention, including informed consent for publication of identifying information and images in an online open-access publication. Infants were between the ages of 4 and 20 weeks. Inclusion criteria included: mothers 18 years of age or more, able to speak and read English without assistance, breastfeeding an infant 1–6 months of age that has not started solid foods.

Four of the volunteer mother-baby pairs reported persisting nipple pain, despite prior comprehensive lactation support. In these pairs, 3 infants had difficulty latching and fussed at the breast, and 3 had been diagnosed with oral ties. The fifth pair was breastfeeding successfully. The cases are described in Table 1.

**Table 1:** Case descriptions

Case	M/F	Gestation	Age (weeks)	Mother’s lactation	Breastfeeding history	Previous lactation support	Pre- and post-intervention milk transfer by test weigh	Direct breastmilk intake/24 h	EBM intake/24 h	Formula vol/24 h
**A**	F	36 + 4	6	Second	Exclusive breastmilk. Maternal nipple pain with breastfeeding from birth. Infant often backarched, cried, and pulled off the breast. Always used nipple shield for both pain and infant behaviour.	Yes	Pre- R breast with and without nipple shield:36 ml. Post- R breast with nipple shield, milk transfer not measured.	Not measured	200 mls	100 mls
B	M	Term	18	Second	Exclusively breastfed, good weight gains, no breastfeeding problems.	No	Pre- L breast: 26 ml. Post- L breast: 14 ml.	540 ml	0	0
C	F	Term	22	First	Severe persistent maternal nipple pain and damage from birth, associated with early suboptimal weight gain which had been recovered. Laser frenotomies of the sublingual and labial frenula at 20 weeks for diagnoses of upper and lower lip-ties and posterior tongue-tie. Post frenotomy, mother reported pain had ‘100% worsened‘, infant behaviour had worsened, and ‘latch was shallow’.	Yes	Pre- L breast: 40 ml. Post - R breast: 55 ml.	Not measured	214 mls	0
D	M	Term	8	First	Breast augmentation 5 years previously, with residual altered sensation and numbness bilaterally, and previous bilateral nipple piercing. Maternal nipple pain in first weeks with suspected low supply. Infant fed EBM every 3 h for the first 4 weeks. Posterior tongue-tie and upper lip-tie diagnosed and referred for frenotomies. Parents didn’t proceed with frenotomies. Saw an IBCLC and baby started to feed from breast. At the time of study, the mother reported persisting difficulty with latching, painful, damaged nipples, oversupply, and vasospasm.	Yes	Pre -R breast: 40 ml. Post- R and L breasts:88 ml	919 mls	0	0
E	M	Term	4	Second	Severe maternal nipple pain from the first breastfeed, ongoing. Tongue-tie and upper lip-tie diagnosed. Parents did not proceed with frenotomy. Infant breastfed intermittently with a nipple-shield, but primarily EBM. Intensive IBCLC support. Infant weight gain normal. Researchers noted a slight anterior membrane that attached to 10% of the ventral surface of the tongue. At time of study, mother offered the breast once a day always with nipple shield; infant would take breast once every few days.		Pre- L breast unable to breastfeed due to unsettled behaviour, use of nipple shield unsuccessful. Given 22 ml EBM from bottle. Post- L breast with nipple shield: 38 ml.	400 mls	Not measured	Formula top ups had recently been introduced.

### Measurement

A sonographer with extensive experience in application of ultrasound to breastfeeding mother-baby pairs applied ultrasound to image tongue positioning and mobility in the five infants during a baseline breastfeed during which infants were placed in their usual position to breastfeed. Ultrasound images were made with a 6 V1/11 endocavity transducer (Sonologic SonoScape S6, Brisbane, Australia). Real time ultrasound images were recorded via Power Lab (ADInstruments) and the software package Chart v5.0.2 (ADInstruments) [48]. A brief gestalt breastfeeding intervention was then delivered [35, 52]. Immediately after application of the intervention, ultrasound imaging was performed. Images were subsequently transferred to a laptop computer. Image measurements were made using ‘Screen Calipers’, V. 4.0 (Iconico Inc. New York, US). The measures used in this study have been validated for intra-rater and inter-rater reliability [45]. The amount of milk consumed during breastfeeds over a 24 h period was determined by test weighing the baby using an electronic balance (Medela Electronic Baby-Weigh Scales, Medela AG, Switzerland) prior to and after breastfeeding [57].

## Results

**Fig. 1:**
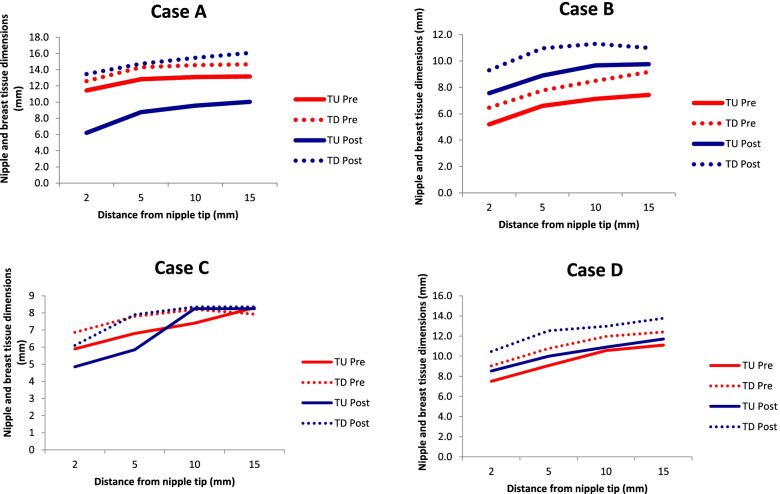
Ultrasound measurements of nipple and breast tissue dimensions pre- and post-intervention

**Fig. 2:**
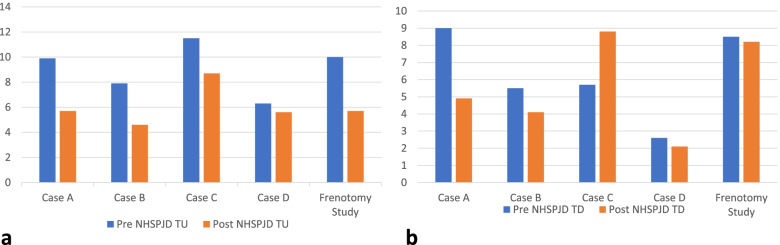
Ultrasound measurements of distance from nipple tip to junction hard and soft palate pre- and post-intervention c.f. frenotomy case report Garbin et al 2013

**Fig. 3:**
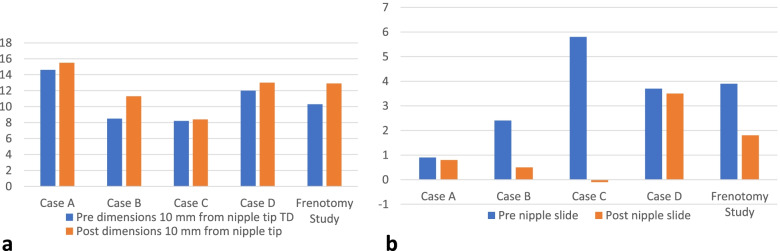
Ultrasound measurements of nipple and breast tissue expansion and nipple slide pre- and post-intervention c.f. frenotomy case report Garbin et al 2013

Table 2 details ultrasound measurements pre- and post-intervention in Cases A and B. Table 3 details ultrasound measurements pre- and post-intervention in Cases C and D. Fig. 1 is a line graph which illustrates nipple and breast tissue width or diameter in tongue up (TU) and tongue down (TD) pre- and post-intervention in Cases A, B, C and D. Fig. 2 is a bar graph which illustrates pre- and post-intervention measures in Cases A, B, C and D at: (a) the distance between the nipple tip and junction of the hard and soft palate (NHSPJD) at tongue up; (b) NHSPJD at tongue down, and compares these changes with NHSPJD findings in a pre- and post-frenotomy case study [58]. Fig. 3 illustrates, in bar graph format, the pre- and post-intervention measures in Cases A, B, C and D at: (a) nipple and breast tissue width or diameter at 10 mm behind tip of nipple; and (b) nipple slide, and compares these changes with measures in a pre- and post-frenotomy case study [58].

**Table 2:** Ultrasound measurements in millimetres pre- and post-intervention in Cases A and B

ULTRASOUND MEASURES	CASE A						***CASE B***					
	Pre TU	Pre TD	Pre-intervention difference between TU and TD	Post TU	Post TD	Post-intervention difference between TU and TD	***Pre TU***	***Pre TD***	***Pre-intervention difference between TU and TD***	***Post TU***	***Post TD***	***Post-intervention difference between TU and TD***
NHSPJD	9.9	9.0	Nipple slide 0.9	5.7	4.9	Nipple slide 0.8	*7.9*	*5.5*	*Nipple slide 2.4*	*4.6*	*4.1*	*Nipple slide 0.5*
IOD	0	5.9		2	5.5	Difference pre & post between IOD at TD −0.4	*0*	*1.8*		*0*	*3*	*Difference pre & post between IOD at TD 1.2*
Nipple and breast tissue dimension (measured at the following distances from nipple tip)												
2	11.4	12.6	1.2	6.2	13.5	7.3	*5.2*	*6.5*	*1.3*	*7.6*	*9.3*	*1.7*
5	12.8	14.3	1.5	8.8	14.7	5.9	*6.6*	*7.8*	*1.2*	*8.9*	*11*	*2.1*
10	13.1	14.6	1.5	9.6	15.5	5.9	*7.1*	*8.5*	*1.4*	*9.7*	*11.3*	*1.6*
15	13.2	14.7	1.5	10	16.1	6.1	*7.4*	*9.2*	*1.8*	*9.8*	*11*	*1.2*
Av.	12.6	14.0	1.4	8.7	15	7.8	*6.6*	*8*	*1.4*	*9*	*10.7*	*1.7*

**Table 3:** Ultrasound measurements in millimetres pre- and post-intervention in Cases C and D

ULTRASOUND MEASURES	CASE C						***CASE D***					
	Pre TU	Pre TD	Pre-intervention difference between TU and TD	Post TU	Post TD	Post-intervention difference between TU and TD	***Pre TU***	***Pre TD***	***Pre-intervention difference between TU and TD***	***Post TU***	***Post TD***	***Post-intervention difference between TU and TD***
NHSPJD	11.5	5.7	Nipple slide 5.8	8.7	8.8	Nipple slide - 0.1	*6.3*	*2.6*	*Nipple slide 3.7*	*5.6*	*2.1*	*Nipple slide 3.5*
IOD	0	1.9		0	1.5	Difference pre & post between IOD at TD - 0.4	*0*	*4*		*0*	*4*	*0*
Nipple and breast tissue dimension (measured at the following distances from nipple tip)												
2	6.1	6.9	0.8	4.9	6.1	1.2	*7.5*	*9.0*	*1.5*	*8.5*	*10.5*	*2.0*
5	6.8	7.8	1.0	5.9	7.9	2.0	*9.0*	*10.8*	*1.8*	*10*	*12.5*	*2.5*
10	6.9	8.2	1.3	8.3	8.4	0.1	*10.6*	*12.0*	*1.4*	*10.9*	*13.0*	*2.1*
15	6.9	7.9	1.0	8.3	8.4	0.1	*11.1*	*12.4*	*1.3*	*11.7*	*13.8*	*2.1*
Av.	6.7	7.7	1.0	6.9	7.7	0.8	*9.5*	*10.5*	*1.5*	*10.3*	*12.5*	*2.2*

**Fig. 4:**
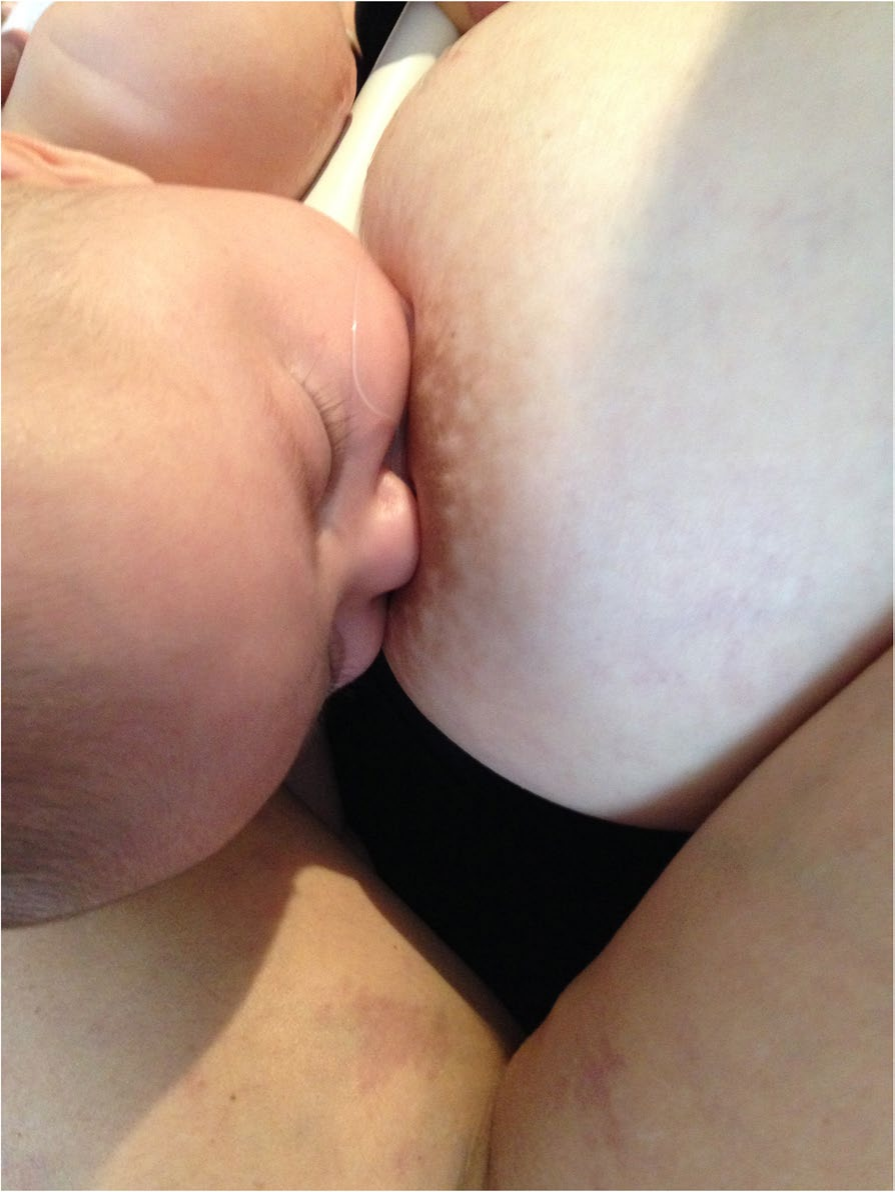
Case A post-gestalt intervention photo 1

**Fig. 5:**
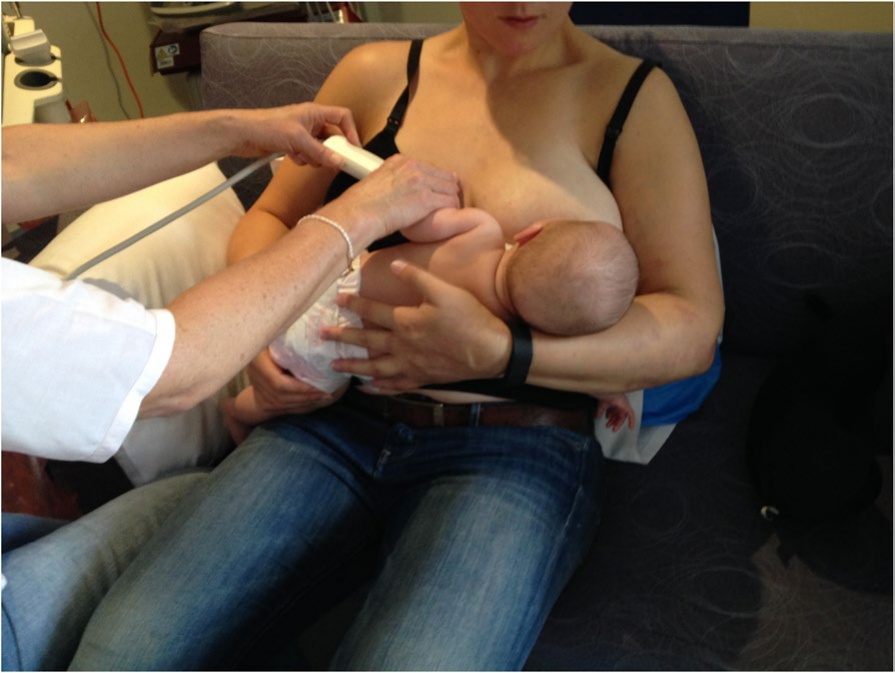
Case A post-gestalt intervention photo 2

Case A comprised a mother and her 6-week-old infant, born 36 + 4 weeks gestation, who was fed only breastmilk either from the breast or as expressed breast milk (EBM) in a bottle. The mother had experienced nipple pain from birth. The baby often backarched, cried, and pulled off the breast when the mother attempted to breastfeed. The mother always used a nipple shield because of pain and fussy infant behaviour. Ultrasound measurements (Table 2 Ultrasound measurements Case A; Figs. 1, 2 and 3) showed decreased distance between the nipple tip and the junction of the hard and soft palate after a brief gestalt intervention, accompanied by increase in nipple and breast tissue dimensions. The amount of nipple slide remained the same. Post-intervention, the mid-tongue no longer opposed the hard palate at the beginning and end of the suck cycle, but rested at a depth of 2 mm, indicating that the pharyngeal seal now occurred more posteriorly. See ‘Fit and hold observations before and after a brief gestalt intervention, illustrated in photographs and videos’, Additional file 2 Case A; ‘Case A Pre-intervention video’, Additional file 3; Fig. 4 Case A post-intervention photo 1; and Fig. 5 Case A post-intervention photo 2.

Case B was a mother with no breastfeeding problems. Her 4.5-month-old exclusively breastfed term infant was gaining weight well. Ultrasound measurements (Table 2 Ultrasound measurements Case B; Figs. 1, 2 and 3) showed that NHSPJD decreased after a brief gestalt intervention, accompanied by increase in nipple and breast tissue dimensions. The amount of nipple slide decreased, and IOD increased at TD. The mother commented: “I felt a difference from when we first started [with the gestalt method]. I felt he was taking more and more breast.”

**Fig. 6:**
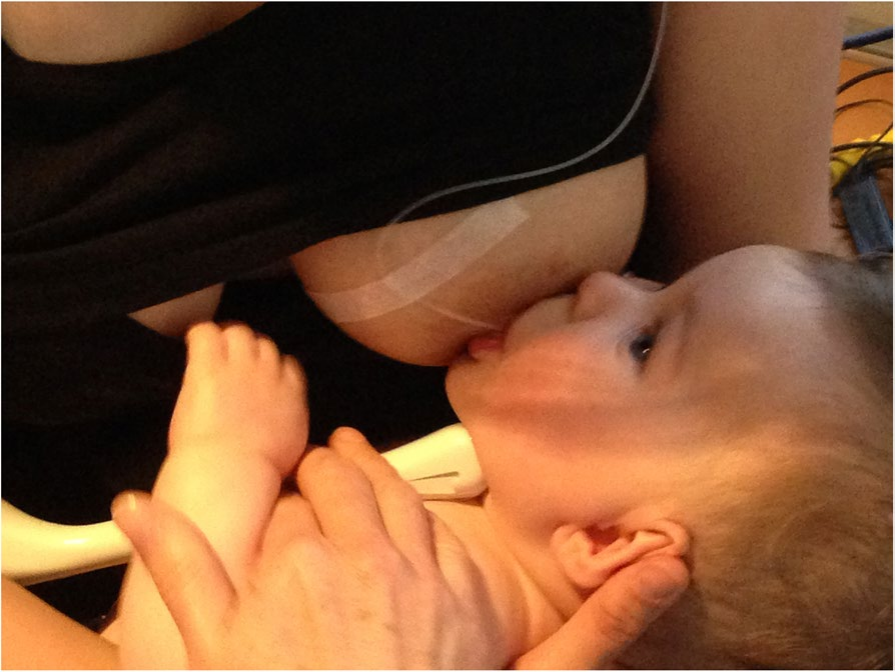
Case C pre-gestalt intervention photo 1

**Fig. 7:**
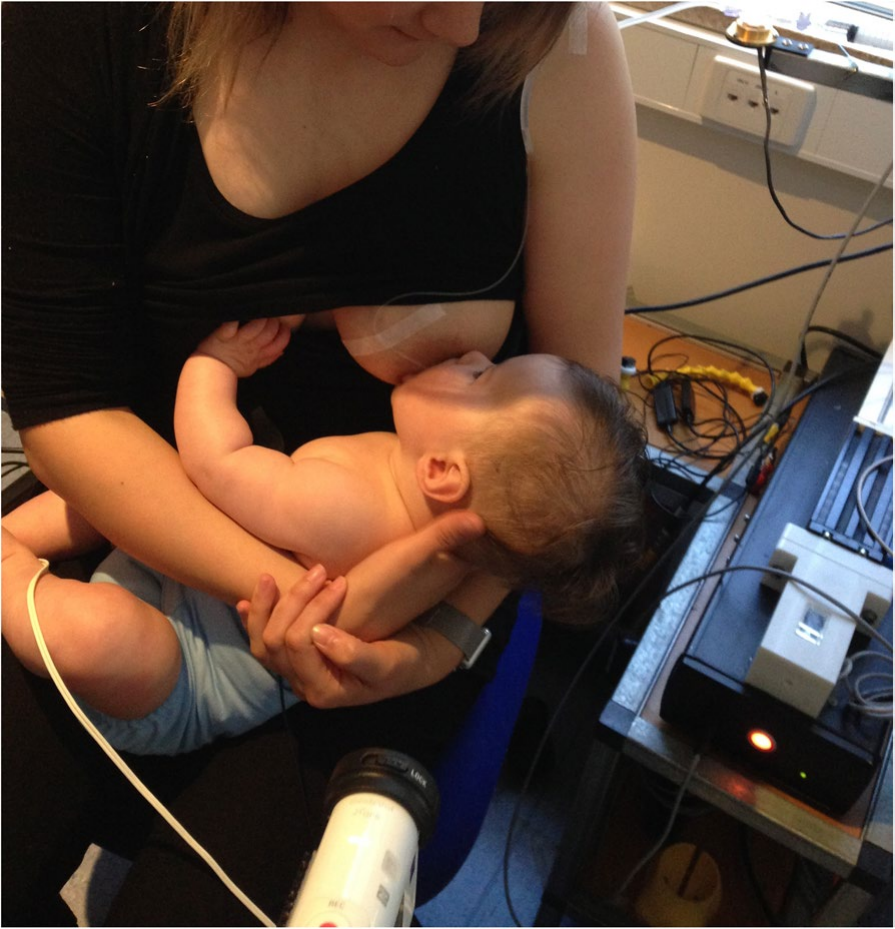
Case C pre-gestalt intervention photo 2

Case C comprised a mother and her 5.5-month-old term baby, who was fed only breast milk. The mother described severe and persistent nipple pain and damage from birth. A fortnight prior to the study, the infant was treated with laser frenotomies of the sublingual and labial frenula. The mother reported “100% worsened nipple pain” since the procedures, associated with what she described as a very shallow latch and worsened fussing and pulling off the breast. Ultrasound measurements (Table 3 Ultrasound measurements Case C; Figs. 1, 2 and 3) showed that NHSPJD decreased after a brief gestalt intervention in TU and increased in TD. Average nipple and breast tissue dimensions did not change, though the nipple tip was narrower and the nipple and breast tissue wider at 15 mm post-intervention. Nipple slide decreased, and IOD slightly decreased at TD. See ‘Fit and hold observations before and after a brief gestalt intervention, illustrated in photographs and videos’, Additional file 2 Case C; Fig. 6 Case C pre-intervention photo 1; and Fig. 7 Case C pre-intervention photo 2.

**Fig. 8:**
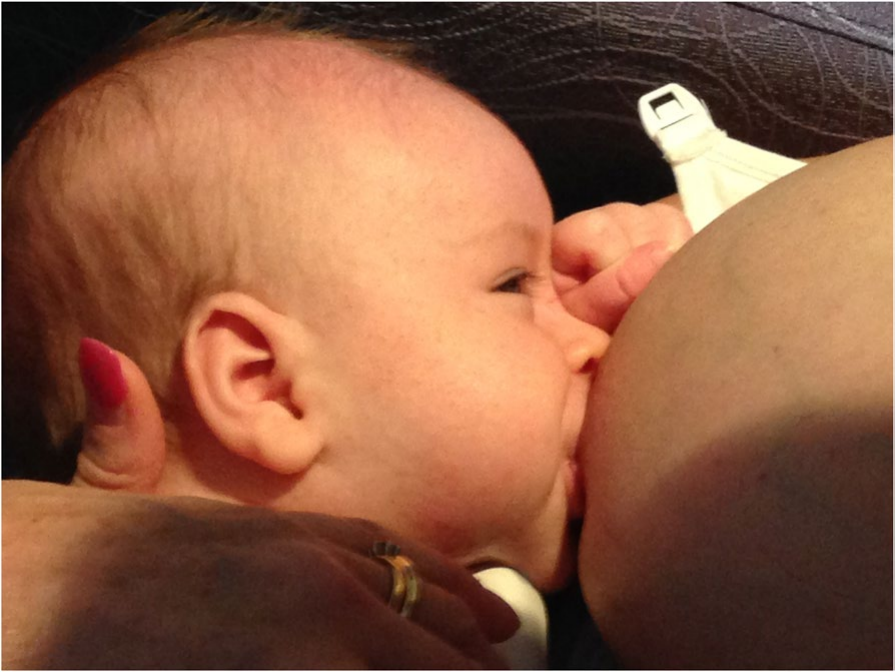
Case D pre-gestalt intervention photo 1

**Fig. 9:**
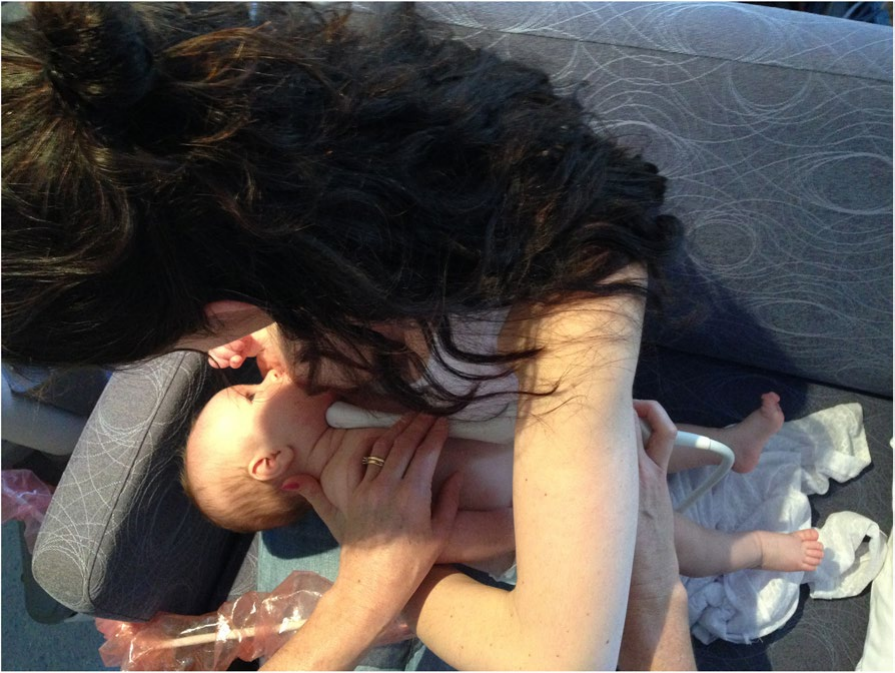
Case D pre-gestalt intervention photo 2

**Fig. 10:**
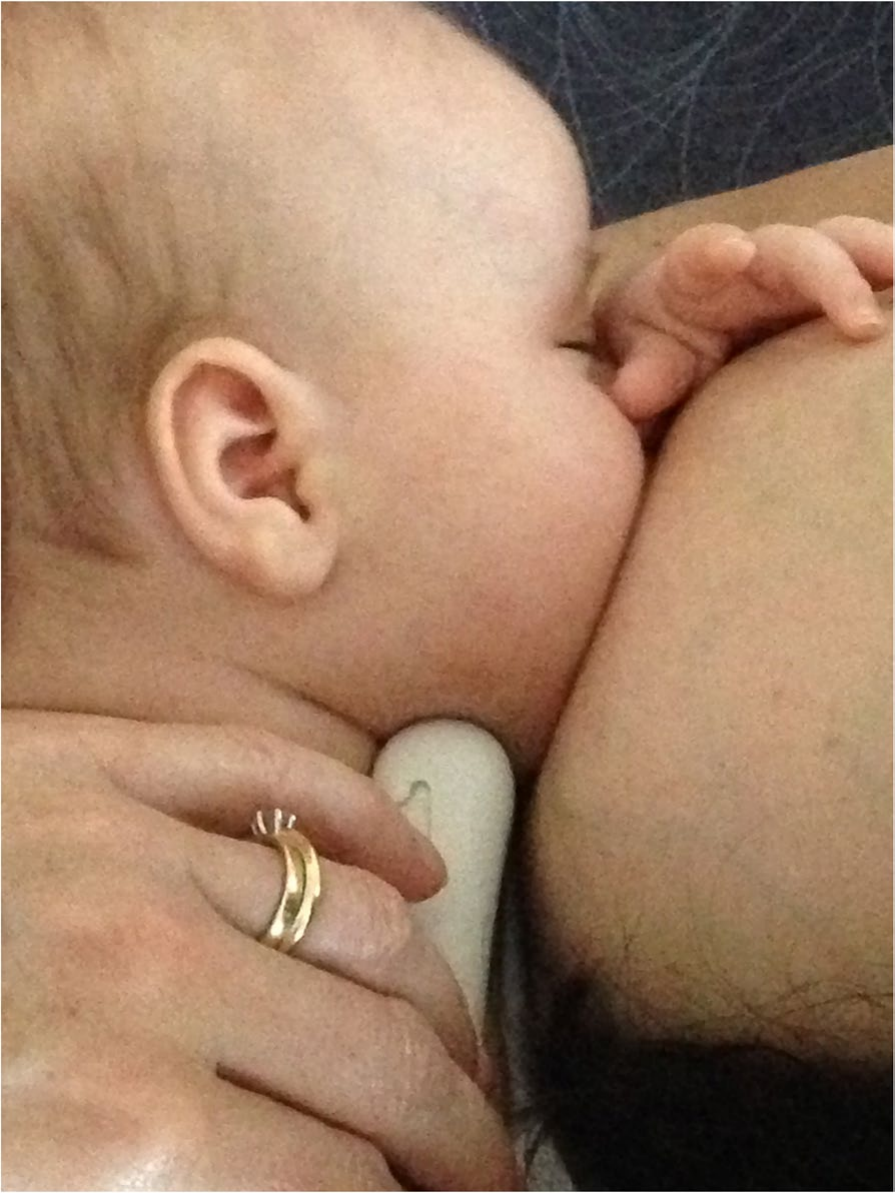
Case D post-gestalt intervention photo 1

**Fig. 11:**
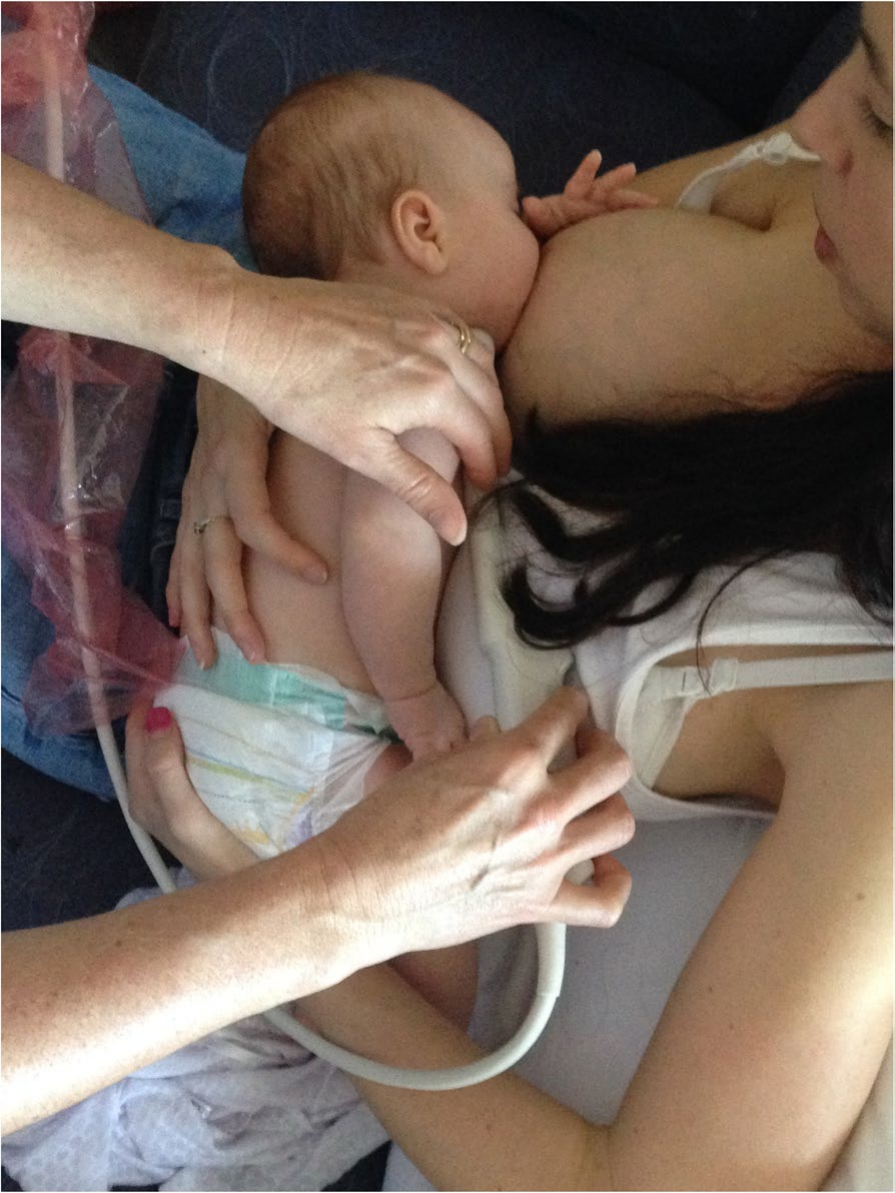
Case D post-gestalt intervention photo 2

Case D comprised a mother and her exclusively breastfed 8-week-old infant. The mother experienced nipple pain and low supply in the first weeks post-birth, and the infant was fed expressed breast milk (EBM) for the first month of life. During this time the infant was diagnosed with ‘posterior tongue-tie’ and ‘upper lip-tie’ but did not receive frenotomies. After comprehensive lactation support, the baby began to feed from the breast. The mother presented for this study reporting persistent difficulty with latching, painful and damaged nipples, and vasospasm. The pair received a very brief gestalt intervention prior to the first ultrasound analysis because the infant became very unsettled in the usual feeding position and was unable to attach to the breast. After the first very brief intervention, the baby latched, and the first ultrasound analysis was performed. A second brief gestalt intervention was then again applied, and post-intervention ultrasound analysis performed. Ultrasound measurements (Table 3 Ultrasound measurements Case D; Figs. 1, 2 and 3) showed that NHSPJD decreased in TU and TD after the second brief gestalt intervention. Nipple and breast tissue dimensions in both tongue up and tongue down increased. The amount of nipple slide decreased, and IOD remained unchanged. See ‘Fit and hold observations before and after a brief gestalt intervention, illustrated in photographs and videos’, Additional file 2 Case D; Fig. 8 Case D pre-intervention photo 1; Fig. 9 Case D pre-intervention photo 2; ‘Case D pre-intervention video’, Additional file 4; Fig. 10 Case D post-intervention photo 1; Fig. 11 Case D post-intervention photo 2; and ‘Case D post-intervention video’, Additional file 5.

**Fig. 12:**
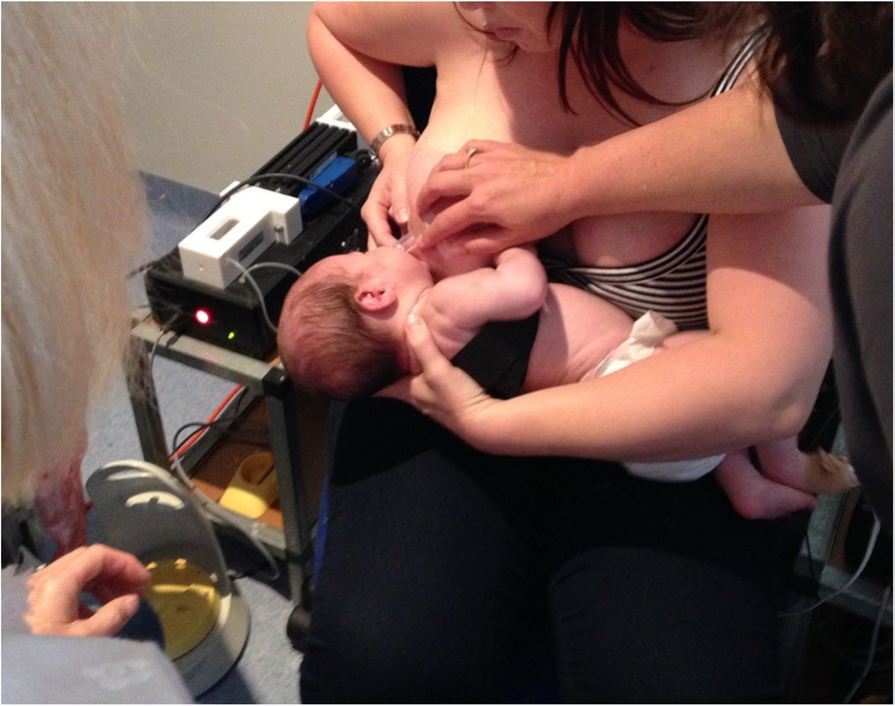
Case E pre-gestalt intervention photo 1

**Fig. 13:**
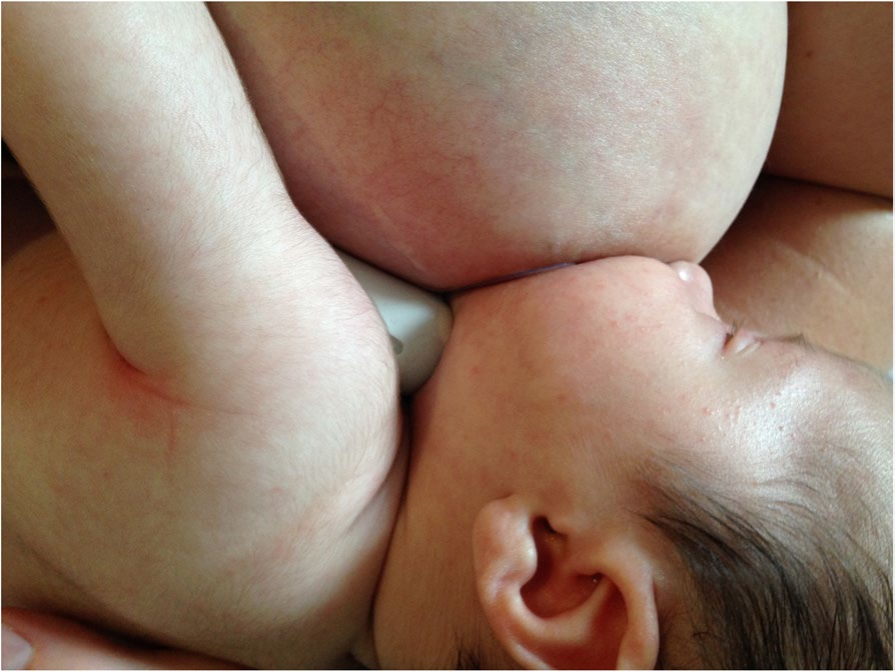
Case E post-gestalt intervention photo 1

**Fig. 14:**
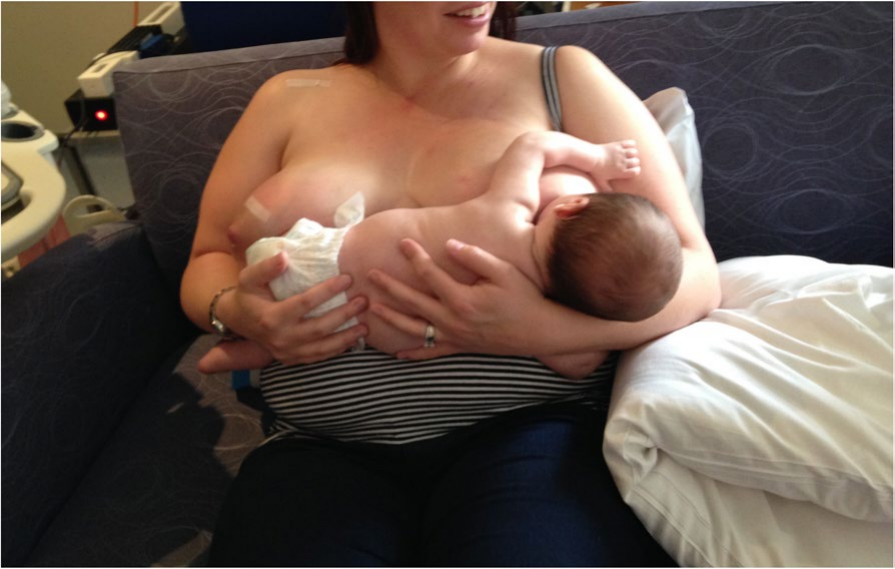
Case E post-gestalt intervention photo 2

**Fig. 15:**
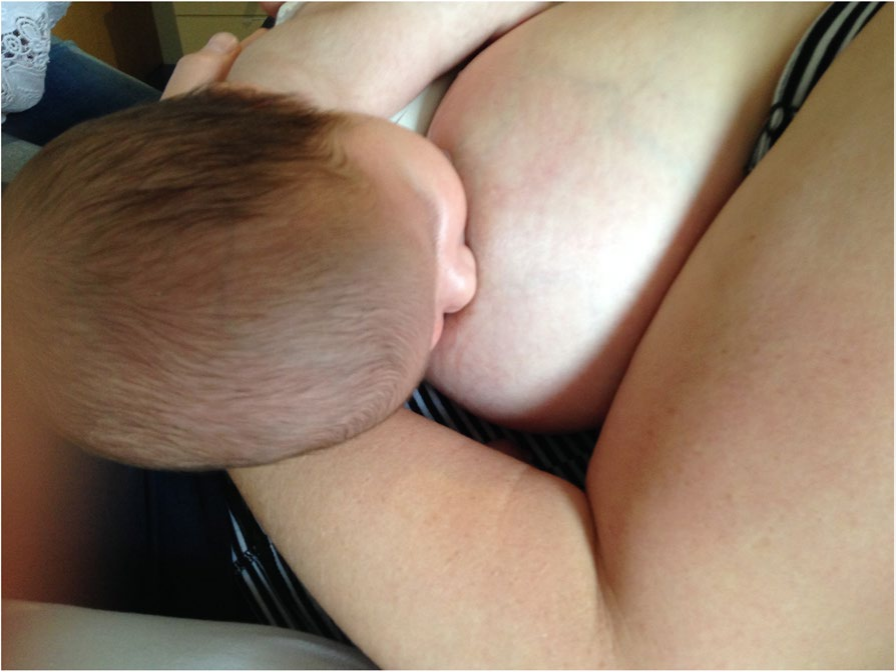
Case E post-gestalt intervention photo 3

Case E comprised a mother and her 4-week-old second born baby, predominantly fed expressed breast milk from a bottle but who had also recently commenced formula top-ups. This mother had experienced severe nipple pain from the first breastfeed, and her infant was diagnosed with tongue-tie and ‘upper lip-tie’ a few days later. The infant had a slight anterior membrane that attached to 10% of the ventral surface of the tongue. The parents did not proceed to frenotomy. The mother had weaned her firstborn at 6 weeks due to fussy behaviour at the breast and was highly motivated to breastfeed this child. She had intensive lactation support from the antenatal period onwards. At the time of the study, the mother was offering the baby the breast once a day, always with a nipple shield to manage pain. The baby would take the breast only once every few days. A pre-intervention ultrasound study was not achieved as the infant could not maintain attachment and was very unsettled at the breast. The baby was then given 22 mls of expressed breast milk and received a standard brief gestalt intervention. Post-intervention the baby settled at the breast and transferred 38 ml. That same evening, the mother emailed a photo of a positionally stable breastfeed as she applied the gestalt method with the nipple shield, and a week later the mother reported by phone that she was now successfully breastfeeding with the nipple shield three times every day. See ‘Fit and hold observations before and after a brief gestalt intervention, illustrated in photographs and videos’, Additional file 2 Case E; Fig. 12 Case E pre-intervention photo 1; ‘Case E pre-intervention video’, Additional file 6; Fig. 13 Case E post-intervention Photo 1; Fig. 14 Case E post-intervention photo 2*,* and Fig. 15 Case E post-intervention photo 3*.*

## Discussion

In summary, ultrasound measurements in four breastfeeding pairs showed that a brief gestalt intervention brought nipple placement closer to the junction of the hard and soft palate, increased nipple and breast tissue dimensions overall, and reduced nipple slide during the suck cycle. Closer nipple placement to the HSPJ and nipple and breast tissue expansion have been interpreted in previous ultrasound studies as signs of improved movement of the tongue relative to the hard palate during sucking. In the gestalt model, these changes are interpreted as signs of increased intra-oral breast tissue volume, to which tongue position and shape conforms. Intra-oral breast tissue volume increases due to elimination of conflicting vectors of force during application of the gestalt intervention [35].

Such changes have been demonstrated in other ultrasound analyses of breastfeeding pairs to be associated with less pain and improved milk transfer [45, 47]. These changes were also observed in the case which didn’t have breastfeeding problems. Anecdotally, breastfeeding is successful in many breastfeeding pairs along a spectrum of breast tissue drag, requiring no intervention. Multiple factors, including more elastic maternal breast tissue or less challenging infant orofacial anatomy, may enhance the resilience of these successful pairs [35]. However, if unsettled infant behaviour or maternal nipple pain emerge, this preliminary study suggests that breast tissue drag may be eliminated and intra-oral breast tissue volume may optimised with a gestalt intervention.

Interestingly, the changes observed by ultrasound in this case series pre- and post-gestalt intervention are congruent with the changes measured by ultrasound pre- and post-frenotomy in a case report by Garbin et al. in 2013 (Table 4; Figs. 2 and 3). In that case, a two-month-old infant was diagnosed with tongue-tie and treated surgically because of a history of poor milk transfer and fussiness at the breast. A palpable band of sublingual connective tissue had been noted on oromotor examination, in the absence of classic tongue-tie [58]. Anatomic dissection clarifies that there is no anatomic basis to the diagnosis of posterior tongue-tie, and a palpable band of sublingual tissue does not indicate abnormality [41]. Comparison between the findings of this preliminary case series and the findings of the 2013 case study suggests that an intervention which aims to help mothers better optimise intra-oral breast tissue volume may obviate the current trend to proceed with surgical and bodywork interventions for diagnoses of oral connective tissue restrictions in the absence of classic tongue-tie.

**Table 4:** Ultrasound measurements in millimetres in pre- and post-frenotomy case (Garbin et al 2013)

ULTRASOUND MEASURES	GARBIN ET AL Pre and Post-Frenotomy Case 2013 [58]					
	Pre TU	Pre TD	Pre-frenotomy difference between TU and TD	Post TU	Post TD	Post-frenotomy difference between TU and TD
NHSPJD	10	6.1	Nipple slide 3.9	5.7	3.9	Nipple slide 1.8
IOD						
Nipple and breast tissue dimension (measured at the following distances from nipple tip)						
2	4.9	8.5	3.6	5.2	8.2	3.0
5	6.9	9	2.1	6.8	10.7	3.9
10	9.8	10.3	0.5	8.6	12.9	4.3
15	–	11.1		9.5	13.7	4.2
Av.	7.2	9.7	2	7.5	11.4	3.9

Nipple pain has been associated with higher baseline and peak vacuums, and the high vacuums found in mothers with pain have been interpreted previously as resulting from abnormal tongue movements [50, 59]. The gestalt model proposes that when a conflicting vector of force or breast tissue drag counters the vacuum generated during sucking, intra-oral vacuum increases because the infant reflexively seeks to maintain optimal distance between the nipple tip and the junction of the hard and soft palate. As a result of this increased vacuum, tensile load may be concentrated on specific parts of the nipple and nipple-areola complex, causing pain and also sometimes damage where the epithelium is most vulnerable (e.g. fissures or cracks on a nipple tip subject to severe stretching loads, or fissures and cracks at base of nipple due to severe stretching loads) [35]. Further interventional studies which measure intra-oral vacuum will be needed to prove this hypothesis. In the gestalt model, nipple slide is conceptualised as a measure of tensile or stretching forces which interact with a woman’s variable breast tissue elasticity, rather than as a measure of harmful friction. Validated pain scores implemented during intervention will serve to determine the contribution of nipple slide to pain experienced during breastfeeding.

A 2015 ultrasound study compared 25 breastfeeding women with persistent nipple pain despite lactation support to 25 controls without nipple pain [50]. Nipple slide from tongue up to tongue down averaged 4.2 mm in the pain group compared to an average of 3.9 mm in those without pain. In our Case A (with pain), the infant always breastfed with a nipple shield in place, which may explain why there is no change in nipple slide post-intervention. In Case B (no pain), the nipple slide decreased from 2.4 to 0.5 post-intervention. In Case C (with pain), the nipple slide dramatically decreased from 5.7 to −0.1 post-intervention. In Case D (with pain), the nipple slide decreased from 3.7 pre-intervention to 2.1 post-intervention.

The 25 mothers experiencing nipple pain showed slightly increased distance between the nipple tip and the junction of the hard and soft palate compared to those without pain [50]. In our Cases A, B, C and D, ultrasound measurements showed that on average, the distance between the nipple tip and the junction of the hard and soft palate decreased 3.2 mm at tongue up and 0.4 mm at tongue down. The 25 mothers experiencing nipple pain also showed reduced expansion of the nipple and breast tissue [50]. In our case series, nipple and breast tissue dimension at 10 mm posterior to the nipple tip increased post-intervention on average by 0.2 mm at tongue up and by 1.2 mm at tongue down, similar to expansion found at 2, 5 and 15 mm from the nipple tip after the gestalt intervention (Fig. 1). The 25 mothers experiencing nipple pain showed a reduced intra-oral depth at full mandibular depression compared to women not experiencing pain [50]. After a brief gestalt intervention, we observed increased intra-oral depth in Case A (with pain) at tongue up only and in Case B (no pain).

The gestalt model proposes that when an infant experiences conflicting vectors of force during breastfeeding, positional instability may result. That is, the infant has difficulty with motoric posture control in the context of breast tissue drag, and signals discomfort. Infant signs of positional instability include difficulty latching, or backarching, fussing, and pulling off during the feed. We applied a very brief gestalt intervention so that one infant (D) was able to maintain positional stability and latch onto the breast for initial analysis. We were unable to collect pre-intervention data in another pair (E), as the infant was initially unable to latch on in the laboratory and had previously only latched on for a breastfeed once every few days, despite prior intensive lactation support. After a feed of 22 mls of EBM and a gestalt intervention, the infant breastfed for post-intervention analysis. The mother reported one week later that since she had been applying the gestalt principles, her infant had been feeding from the breast three times daily.

In the absence of measures from other intervention studies to improve fit and hold (or positioning and attachment) to the breast we made a comparison to a frenotomy case study. Similar findings were observed, with nipple placement improved, nipple slide decreased, and nipple/breast tissue dimensions increased [58]. Studies however are required not only to determine if the gestalt method improves nipple pain but also whether it reduces the need for frenotomy.

## Limitations

Although our case series suggests that a brief gestalt fit and hold intervention impacts on nipple placement and intra-oral nipple and breast tissue expansion during breastfeeding, it is limited by the absence of measures of pain or infant behaviour at the breast, and of short- or long-term changes in breastfeeding success. We cannot discount the possibility that the ultrasound probe alters the way a baby fits into the maternal body, potentially generating new intra-oral vectors of force, though we believe this effect was minimised by our sonographer’s high degree of skill and experience with breastfeeding pairs. In addition, ultrasound analysis delivers measurements in two dimensions. The implications of these measurements for real-world, three-dimensional understandings of breastfeeding biomechanics and clinical applications are interpretative.

## Conclusion

This preliminary study suggests that the gestalt method changes nipple position, increases nipple and breast tissue dimensions, and decreases nipple slide in both a successfully breastfeeding pair and in four mothers who had already received comprehensive lactation support. These encouraging results support the hypothesis that the gestalt approach to fit and hold increases intra-oral breast tissue volume by eliminating conflicting vectors of force. Further research comparing outcomes between the gestalt method and other fit and hold approaches is required.

## Supplementary Information

**Additional file 1.** Key elements of the gestalt approach to clinical breastfeeding support. Step-by-step description of major elements of the gestalt method

**Additional file 2.** Fit and hold observations before and after a brief gestalt intervention, illustrated in photographs and videos. Description of key observations in photographs and videos before and after a brief gestalt intervention

**Additional file 3.** Case A Pre-intervention video. Video taken prior to a brief gestalt intervention

**Additional file 4.** Case D Pre-intervention video. Video taken after a very brief intervention (because mother was unable to bring infant to the breast), but prior to receiving a standard brief gestalt intervention

**Additional file 5.** Case D Post-intervention video. Video taken after standard brief gestalt intervention

**Additional file 6.** Case E Pre-intervention video. Video taken prior to brief gestalt intervention

## Data Availability

All data is available for perusal and is kept securely by the Geddes Hartmann Human Lactation Research Group, The University of Western Australia. The datasets used and analysed during this study are available from the corresponding author on reasonable request.

## References

[1] Odom E, Scanlon K, Perrine C, Grummer-Strawn L (2013). Reasons for earlier than desired cessation of breastfeeding. Pediatics..

[2] Li R, B S, Chen J, Grummer-Strawn LM. (2008). Why mothers stop breastfeeding: mothers' self-reported reasons for stopping during the first year. Pediatrics.

[3] Ayton JE, Tesch L, Hansen E (2019). Women's experiences of ceasing to breastfeed: Australian qualitative study. BMJ Open.

[4] Brown CR, Dodds L, Legge A, Bryanton J, Semenic S (2014). Factors influencing the reasons why mothers stop breastfeeding. Can J Public Health.

[5] Buck ML, Amir LH, Cullinane M, Donath SM (2014). CASTLE study team. Nipple pain, damage and vasospasm in the first eight weeks postpartum. Breastfeed Med.

[6] Dias CC, Figueiredo B (2015). Breastfeeding and depression: a systematic review of the literature. J Affect Disord.

[7] Brown A, Rance J, Bennett P (2016). Understanding the relationship between breastfeeding and postnatal depression: the role of pain and physical difficulties. J Adv Nurs..

[8] Schmied V, Beake S, Sheehan A, McCourt C, Dykes F (2011). Women's perceptions and experiences of breastfeeding support: a metasynthesis. Birth..

[9] Australian National Breastfeeding Strategy (2019). Australian National Breastfeeding Strategy: 2019 and beyond.

[10] Schafer R, Watson GC (2015). Physiologic breastfeeding: a contemporary approach to breastfeeding initiation. J Midwifery Womens Health.

[11] Colson SD, Meek JH, Hawdon JM (2008). Optimal positions for the release of primitive neonatal reflexes stimulating breastfeeding. Early Hum Dev.

[12] Smillie CM, Watson CG (2016). How infants learn to feed: a neurobehavioral model. Supporting sucking skills in breastfeeding infants.

[13] Moore ER, Berman N, Anderson GC, Medley N. Early skin-to-skin contact for mothers and their healthy newborn infants. Cochrane Database Syst Rev. 2016(Issue 11. Art. No.: CD003519. 10.1002/14651858.CD14003519.pub14651854. 10.1002/14651858.CD003519.pub4 PMC6464366 27885658

[14] Labarere J, Bellin V, Fourny M, Gagnaire J-C, Francois P, Pons J-C (2003). Assessment of a structured in-hospital educational intervention addressing breastfeeding: a prospective randomised open trial. BJOB..

[15] Wallace LM, Dunn OM, Alder EM, Inch S, Hills RK, Law SM (2006). A randomised-controlled trial in England of a postnatal midwifery intervention on breast-feeding duration. Midwifery..

[16] Kronborg H, Maimburg RD, Vaeth M (2012). Antenatal training to improve breast feeding: a randomised trial. Midwifery..

[17] Henderson A, Stamp G, J P. (2001). Postpartum positioning and attachment education for increasing breastfeeding: a randomized trial. Birth..

[18] Forster D, McLachlan H, Lumley J, Beanland C, Waldenstrom U, Amir L (2004). Two mid-pregnancy interventions to increase the initiation and duration of breastfeeding: a randomized controlled trial. Birth..

[19] De Oliveira LD, Giugliani ERJ, do Espirito Santo LC. (2006). Effect of intervention to improve breastfeeding technique on the frequency of exclusive breastfeeding and lactation-related problems. J Hum Lact.

[20] Kronborg H, Vaeth M (2009). How are effective breastfeeding technique and pacifier use related to breastfeeding problems and breastfeeding duration?. Birth..

[21] Wood N, K, Woods NF, Blackburn ST, Sanders EA. (2016). Interventions that enhance breastfeeding initiation, duration and exclusivity: a systematic review. MCN..

[22] Ingram J, Johnson D, Greenwood R (2002). Breastfeeding in Bristol: teaching good positioning, and support from fathers and families. Midwifery..

[23] Thompson RE, Kruske S, Barclay L, Linden K, Gao Y, Kildea SV (2016). Potential predictors of nipple trauma from an in-home breastfeeding programme: a cross-sectional study. Women Birth.

[24] Wang Z, Liu Q, Min L, Mao X (2021). The effectiveness of laid-back position on lactation related nipple problems and comfort: a meta-analysis. BMC Pregnancy Childbirth.

[25] Milinco J, Travan L, Cattaneo A, Knowles A, Sola VM, Causin E (2020). Effectiveness of biological nurturing on early breastfeeding problems: a randomized controlled trial. Int Breastfeed J.

[26] Yin C, Su X, Liang Q, Ngai FW. Effect of baby-led self-attachment breastfeeding technique in the postpartum period on breastfeeding rates: a randomized study. Breastfeed Med. 2021. 10.1089/bfm.2020.0395. 10.1089/bfm.2020.0395 33913745

[27] Svensson KE, Velandia M, Matthiesen A-ST, Welles-Nystrom BL, Widstrom A-ME (2013). Effects of mother-infant skin-to-skin contact on severe latch-on problems in older infants: a randomized trial. Int Breastfeed J.

[28] Mitre E, Susi A, Kropp LE, Schwartz DJ, Gorman GH, Nylund CM. Association between use of acid-suppressive medications and antibiotics during infancy and allergic diseases in early childhood. JAMA Pediatr. 2018. 10.1001/jamapediatrics.2018.0315. 10.1001/jamapediatrics.2018.0315 PMC6137535 29610864

[29] Douglas PS (2005). Excessive crying and gastro-oesophageal reflux disease in infants: misalignment of biology and culture. Med Hypotheses.

[30] Douglas P (2013). Diagnosing gastro-oesophageal reflux disease or lactose intolerance in babies who cry alot in the first few months overlooks feeding problems. J Paediatr Child Health.

[31] Gieruszczak-Bialek D, Konarska Z, Skorka A, Vandenplas Y, Szajewska H (2015). No effect of proton pump inhibitors on crying and irritability in infants: systematic review of randomized controlled trials. J Pediatr.

[32] Greenhawt M (2016). Early allergen introduction for preventing development of food allergy. JAMA..

[33] Ierodiakonou D, Garcia-Larsen V, Logan A, Groome A. Timing of allergenic food introduction to the infant diet and risk of allergic or autoimmune disease. JAMA. 2016;316. 10.1001/jaja.2016.12623. 10.1001/jama.2016.12623 27654604

[34] Gordon M, Biagioli E, Sorrenti M, Lingua C, Moja L, Banks SS, et al. Dietary modifications for infantile colic. Cochrane Database Syst Rev. 2018. 10.1002/14651858.CD14601129.pub14651852. 10.1002/14651858.CD011029.pub2 PMC6394439 30306546

[35] Douglas PS, Geddes DB (2018). Practice-based interpretation of ultrasound studies leads the way to less pharmaceutical and surgical intervention for breastfeeding babies and more effective clinical support. Midwifery..

[36] Joseph KS, Kinniburg B, Metcalfe A, Raza N, Sabr Y, Lisonkova S (2016). Temporal trends in ankyloglossia and frenotomy in British Columbia, Canada, 2004-2013: a population-based study. CMAJ Open.

[37] Kapoor V, Douglas PS, Hill PS, Walsh L, Tennant M (2018). Frenotomy for tongue-tie in Australian children (2006–2016): an increasing problem. MJA..

[38] Hale M, Mills N, Edmonds L, Dawes P, Dickson N, Barker D, et al. Complications following frenotomy for ankyloglossia: a 24-month prospective New Zealand Paediatric Surveillance Unit study. J Paediatr Child Health. 2019. 10.1111/jpc.14682. 10.1111/jpc.14682 31714639

[39] Razdan R, Callaham SS, R, Chafin M, Carr MM. Maxillary frenulum in newborns: associations with breastfeeding. Otolaryngol Head Neck Surg. 2020. 10.1177/0194599820913605. 10.1177/0194599820913605 32204658

[40] Ellehauge E, Schmidt Jensen J, Gronhoj C, Hjuler T (2020). Trends of ankyloglossia and lingual frenotomy in hospital settings among children in Denmark. Danish Medical Journal.

[41] Mills N, Keough N, Geddes DT, Pransky S (2019). Defining the anatomy of the neonatal lingual frenulum. Clin Anat.

[42] Douglas PS, Cameron A, Cichero J, Geddes DT, Hill PS, Kapoor V, et al. Australian Collaboration for Infant Oral Research (ACIOR) Position Statement 1: Upper lip-tie, buccal ties, and the role of frenotomy in infants. Australas Dental Pract. 2018:144–6.

[43] Wei E, Tunkel D, Boss E, Walsh J. Ankyloglossia: update on trends in diagnosis and management in the United States, 2012–2016. Otolaryngol Head Neck Surg. 2020. 10.1177/0194599820925415. 10.1177/0194599820925415 32427523

[44] Prevost P, Gleberzon B, Carleo B, Anderson K, Cark M, Pohlman KA. Manual therapy for the pediatric population: a systematic review. BMC Complement Altern Med. 2019. 10.1186/s12906-12019-12447-12902. 10.1186/s12906-019-2447-2 PMC6417069 30866915

[45] McClellan H, Sakalidis VS, Hepworth AR, Hartmann PE, Geddes DB (2010). Validation of nipple diameter and tongue movement measurements with B-mode ultrasound during breastfeeding. Ultrasound Med Biol.

[46] Sakalidis VS, Williams TM, Garbin CP, Hepworth AR, Hartmann PE, Paech MJ (2013). Ultrasound imaging of infant sucking dynamics during the establishment of lactation. J Hum Lact.

[47] Geddes DT, Sakalidis VS. Ultrasound imaging of breastfeeding - a window to the inside: methodology, normal appearances, and application. J Hum Lact. 2016. 10.1177/0890334415626152. 10.1177/0890334415626152 26928319

[48] Geddes DT, Kent JC, Mitoulas LR, Hartmann PE (2008). Tongue movement and intra-oral vacuum in breastfeeding infants. Early Hum Dev.

[49] Elad D, Povlovsky P, Blum O, Laine AF, Po MJ, Botzer E (2014). Biomechanics of milk extraction during breast-feeding. Proc Natl Acad Sci U S A.

[50] McClellan HL, Kent JC, Hepworth AR, Hartmann PE, Geddes DT (2015). Persistent nipple pain in breastfeeding mothers associated with abnormal infant tongue movement. Int J Environ Res Public Health.

[51] Mills N, Pranksky S, Geddes DT, Mirjalili SA. What is a tongue tie? Defining the anatomy of the in-situ lingual frenulum. Clin Anat. 2019. 10.1002/ca.23343. 10.1002/ca.23343 PMC6850428 30701608

[52] Douglas PS, Keogh R (2017). Gestalt breastfeeding: helping mothers and infants optimise positional stability and intra-oral breast tissue volume for effective, pain-free milk transfer. J Hum Lact.

[53] Geddes DB (2007). The anatomy of the lactating breast: latest research and clinical implications. Infant..

[54] Ramsay DT, Kent JC, Hartmann RA, Hartmann PE (2005). Anatomy of the lactating human breast redefined with ultrasound imaging. J Anat.

[55] Mills N, Lydon A-M, Davies-Payne D, Keesing M, Geddes DT, Mirjalili SA (2020). Imaging the breastfeeding swallow utilising real-time MRI.

[56] Sanuki J, Fukuma E, Uchida Y (2009). Morphologic study of nipple-areola complex in 600 breasts. Aesthet Plast Surg.

[57] Arthur PG, Hartmann PE, Smith M (1987). Measurement of milk intake of breastfed infants. J Pediatr Gastroenterol Nutr.

[58] Garbin CP, Vs S, Chadwick LM, Whan E, Hartmann PE, Geddes DT (2013). Evidence of improved milk intake after frenotomy: a case report. Pediatrics..

[59] McClellan HI, Geddes DT, Kent JC, Garbin CP, Mitoulas LR, Hartmann PE (2008). Infants of mothers with persistent nipple pain exert strong sucking vacuums. Acta Paediatr.

