# Supplementary material for: A brief gestalt intervention changes ultrasound measures of tongue movement during breastfeeding: case series
Source: BMC Pregnancy Childbirth. 2022 Feb 1;22:94. doi: 10.1186/s12884-021-04363-7 (PMC8808964; doi:10.1186/s12884-021-04363-7)
Supplement: Supplementary file 1 — Additional file 1. Key elements of the gestalt approach to clinical breastfeeding support. Step-by-step description of major elements of the gestalt method [file 12884_2021_4363_MOESM1_ESM.docx]

**Additional File 1**

**Key elements of the gestalt approach to clinical breastfeeding support**

**Overview of the gestalt method**

Adapted from

[Douglas PS, Keogh R. Gestalt breastfeeding: helping mothers and infants optimise positional stability and intra-oral breast tissue volume for effective, pain-free milk transfer. *Journal of Human Lactation*. 2017;33(3):509–518.](https://www.researchgate.net/publication/317595543_Gestalt_Breastfeeding_Helping_Mothers_and_Infants_Optimize_Positional_Stability_and_Intraoral_Breast_Tissue_Volume_for_Effective_Pain-Free_Milk_Transfer)^1^

The gestalt method integrates four theoretical frames:

1. Complexity or dynamic systems science. (The mother-baby pair are a complex adaptive system. The gestalt intervention supports adaptivity, resilience, and empowerment when problems emerge, so that perturbed feedback loops can be dampened down through maternal experimentation)^2^
2. Physiological breastfeeding initiation (also known as laid-back breastfeeding or biological nurturing)^3-7^
3. Gestalt model of the biomechanics of infant suck, which has been elucidated in ultrasound, vacuum and MRI studies^1, 8, 9^
4. Psychological strategies from applied functional contextualism, popularly known as Acceptance and Commitment Therapy, which includes empowering a woman to attend mindfully to sensations within her own body, breast and nipple, and also her baby’s subtle communications or cues whilst feeding.^10-13^

In breastfeeding, a woman’s unique anatomy, including breast size and elasticity; abdominal shape; lap length; nipple shape, size and direction; and upper and forearm length and shape, and her baby’s unique anatomy, including palate contour; oral connective tissue attachments and elasticities; tongue length; oral cavity size; and chin shape need to fit together in a way that supports positional stability, nipple protection or healing, and optimal milk transfer. This is particularly important in mother-baby pairs facing anatomic challenges, including (but not limited to) inelastic breast tissue, generous or pendulous breasts, recessed infant mandible, high infant palate, and maternal obesity.

Some babies breastfeed without difficulty despite substantial breast tissue drag and require no intervention. However, nipple pain, difficulty latching on, fussing and pulling off the breast, backarching, or apparent refusal to feed are typical signs of breast tissue drag. Positional instability results in suboptimal intra-oral breast tissue volume, which can result in poor milk transfer and nipple pain.^8^

A gestalt intervention is delivered flexibly in response to a woman and her baby’s needs, so that the mother is empowered to experiment and discover a fit and hold which works for her and her baby across their unique anatomic configurations. The gestalt method proposes that all women have the right to be educated in:

1. Laid-back or biological nurturing principles
2. Strategies which optimize the biomechanics of suck and eliminate breast tissue drag e.g. by optimizing cervical spine extension, spinal alignment, symmetrical face-breast bury, and healthy symmetrical oromotor and musculoskeletal function
3. Simple psychological strategies for managing the difficult thoughts and feelings which often accompany breastfeeding difficulty.

The gestalt method avoids ideology and uses a set of carefully selected words and terms which are non-mechanistic and emotionally neutral or encouraging.^14^ Many techniques popularly used in clinical breastfeeding support are not used in gestalt breastfeeding, because they interfere with the baby’s breastfeeding reflexes and intra-oral breast tissue volumes. Lists of some terms used and of popular approaches to breastfeeding fit and hold which do not belong to the gestalt method are detailed in Douglas and Keogh 2017.^1^

In the gestalt method, there are three pillars to positional stability:

1. Absence of nipple and breast pain,
2. Infant stable at breast, that is, is not having difficulty latching, or back arching, fussing or pulling off at the breast,
3. Superior and lateral views of the face-breast bury are symmetrical, and a frontal view shows spinal alignment (no twisting of pelvis relative to head)

A brief intervention focusses on communicating an overview of the key strategies of a gestalt intervention, delivered in a five to ten-minute time interval. A comprehensive gestalt intervention may take between twenty and forty-five minutes, with follow-ups as required.

**Key steps of a gestalt intervention**

**Step 1. Education about gestalt biomechanics of suckling, developed from ultrasound, vacuum and MRI studies of breastfeeding mothers and babies**

***A.Intra-oral vacuum***

A demonstration video (<https://possumsonline.com/video/baby-transfers-milk-beautifully>) illustrates what healthy infant suck looks like during letdown. The breastfeeding support professional (BSP) uses a knitted breast and her fingers to illustrate how infant’s jaw drops, which creates a vacuum.

***B. Symmetrical face-breast bury***

The same demonstration video is also used to illustrate

- Symmetrical face-breast bury, from the mother’s view
- Nose is resting against the breast but the infant breathing freely
- No visible lips.

A demonstration photograph is used to illustrate the symmetrical face-breast bury from the lateral view.

***C. Intra-oral breast tissue volume***

A knitted breast and the BSP’s fingers are used to illustrate how the jaw drops reflexly, and the vacuum which is created incrementally daws up more and more breast tissue until the baby’s mouth is wide open. That can’t be seen though because the lower half of the face and lips are deeply buried. If the lips are visible, there will be breast tissue drag.

***D. Landing pad (description)***

The BSP uses the knitted breast to illustrate how, in order to achieve a symmetrical face-breast bury, an area of the breast approximately 10 centimetres in diameter (or 5 cm radius symmetrically around the nipple) needs to be exposed.

***E. Breast tissue drag***

The BSP uses the knitted breast to illustrate how

- Breast and nipple, when exposed to gravity, fall into a position unique to that woman, and
- If there is breast tissue drag, the nipple and areolar complex are dragged in a direction which conflicts with the direction of the intra-oral vacuum.

**Step 2. The woman prepares her body and mind for breastfeeding (laid-back or biological nurturing approach to breastfeeding, integrated with Acceptance and Commitment Therapy)**

***A. Deck-chair position (laid-back breastfeeding or biological nurturing)***

Invite the woman to lie back in the chair at an angle of 45 degrees or so, ensuring that she has plenty of space on either side of her body (not impacted by the arms of breastfeeding chairs). When at home, a low footstool is helpful.

***B. Landing pad (eliminate encroachments)***

Ensure that the landing pad is not compromised, by

- Bra or garments
- Mother’s upper arm pressed against baby’s forehead
- Mother’s abdomen

***C. Acceptance and Commitment Therapy strategies***

If the woman has nipple pain but wants to continue bringing the baby to the breast, invite:

- Deep breathing
- Conscious muscle relaxation especially of the shoulders and arms

***D. When out and about***

- Workability not perfection
- ‘Do what you can’
- ‘You can be slumped back to breastfeed when you are out and no-one would know’

**Step 3. Switching on an infant’s mammalian reflexes (laid-back breastfeeding or biological nurturing)**

1. Infant chest and abdomen are flat against mother’s torso.
2. Infant hands are bare and explore mother’s breast. Hands and arms are never tucked between the mother and baby’s body.
3. Baby’s head is supported on the mother’s ipsilateral forearm. The baby is placed face-down into breast above the nipple and areolar complex, which orients the baby.
4. This triggers oral rooting reflex. The mother uses her forearm, with ipsilateral hand and wrist relaxed and not flexed tightly in against the baby, as a lever to help the baby on, mouth over the nipple. Instructions such as nipple to nose, wait for wide gape, or take baby off if ‘not on deep’ are unhelpful and are not used. Babies typically come on to the breast shallowly but micro-movements, as the baby’s jaw drops reflexly, will ensure that optimal intra-oral breast tissue volume is achieved.
5. Ribcage wrap if newborn or still small enough, by securing baby’s trunk, pelvis, hips, and legs up under the woman’s other breast, lifting up breast if necessary. This ensures optimal cervical spine extension and excellent spinal alignment for good oromotor function. Older, longer babies are placed more diagonally across mother’s reclined torso, with chest and abdomen still firmly pressed against mother’s body, and infant’s lower hip resting on mother’s upper thigh.
6. Create a ‘paddle hand’ (fingers held together, palm flat) to place firmly between infant’s shoulder blades. The woman does not need to carry baby’s weight if semi-reclined, as the paddle hand stabilises baby against her body and supports optimal cervical extension.

**Step 4. Micro-movements (derived from the gestalt biomechanical model)**

***A. Three directions***

Micro-movements are movements of one or two millimetres magnitude only. They are controlled by the mother’s forearm, which is placed under the baby’s head, in each of three directions:

- - Vertical
  - Horizontal
  - A forearm roll to control the angle of the face breast bury

The clinician asks for consent to place her hands on the mother’s forearm and supports gentle experimentation with micro-movements, in order to facilitate somatic learning (or to lay down new neural pathways in the maternal brain).

***B. Breast and nipple sensation***

Mother invited to feedback degree of nipple discomfort or pain. The clinician may use the Numerical Rating Scale of 0-10 (where 10 is most severe pain possible), by supporting micro-movements and asking for a rating after each micro-movement on the pain scale. Some women may prefer to simply respond to the questions: Is that better? Is that worse?

***C. Baby’s cues***

The mother and clinician observe the baby’s communications, even very subtle sounds and expressions, and respond with micro-movements until the baby is deeply relaxed and stable in the breastfeed.

***D. Three visual indicators of positional stability***

Check out the three views for symmetry and alignment:

- - Superior view of symmetrical face-breast bury
  - Lateral view of symmetrical face-breast bury
  - Frontal view of alignment of baby’s spine

***E. Three phone photos***

Inquire if parents would like three photos taken on their phone of these three views, to take home.

**More detailed information about delivering a gestalt breastfeeding intervention**

1. A detailed online program with videos and photographs is available at: <https://education.possumsonline.com/programs/gestalt-breastfeeding-online-program>
2. Health professionals including International Board Certified Lactation Consultants are able to upskill in the gestalt method of clinical breastfeeding support by participating in Neuroprotective Developmental Care (NDC) Accreditation. NDC is an evolutionarily aligned evidence-based approach to infant-care across the domains of breastfeeding, cry-fuss problems, sleep and maternal mood. NDC builds upon breastfeeding as the biological foundation for maternal-infant interaction. NDC Accreditation provides detailed consultation templates and assessment tools for application of the gestalt method. NDC Accreditation is available at <https://education.possumsonline.com/accreditation>

**References**

1. Douglas PS, Keogh R. Gestalt breastfeeding: helping mothers and infants optimise positional stability and intra-oral breast tissue volume for effective, pain-free milk transfer. *Journal of Human Lactation*. 2017;33(3):509–518.

2. Douglas PS, Hill PS, Brodribb W. The unsettled baby: how complexity science helps. *Arch Dis Child*. 2011;96:793-797.

3. Wang Z, Liu Q, Min L, Mao X. The effectiveness of laid-back position on lactation related nipple problems and comfort: a meta-analysis. *BMC Pregnancy and Childbirth*. 2021;21:248.

4. Yin C, Su X, Liang Q, Ngai FW. Effect of baby-led self-attachment breastfeeding technique in the postpartum period on breastfeeding rates: a randomized study. *Breastfeeding Medicine*. 2021:doi:10.1089/bfm.2020.0395.

5. Milinco J, Travan L, Cattaneo A, Knowles A, Sola VM, Causin E, et al. Effectiveness of biological nurturing on early breastfeeding problems: a randomized controlled trial. *International Breastfeeding Journal*. 2020;15(1):21.

6. Schafer R, Watson Genna C. Physiologic breastfeeding: a contemporary approach to breastfeeding initiation. *Journal of Midwifery and Women's Health*. 2015;60:546-553.

7. Colson SD, Meek JH, Hawdon JM. Optimal positions for the release of primitive neonatal reflexes stimulating breastfeeding. *Early Hum Dev*. 2008;84:441-449.

8. Douglas PS, Geddes DB. Practice-based interpretation of ultrasound studies leads the way to less pharmaceutical and surgical intervention for breastfeeding babies and more effective clinical support. *Midwifery*. 2018;58:145–155.

9. Mills N, Lydon A-M, Davies-Payne D, Keesing M, Mirjalili SA, Geddes DT. Imaging the breastfeeding swallow: pilot study utilizing real-time MRI. *Laryngoscope Investigative Otolaryngology*. 2020;5:572-579.

10. Whittingham K, Douglas PS. "Possums": building contextual behavioural science into an innovative evidence-based approach to parenting support in early life. In: Kirkaldy B, editor. Psychotherapy in parenthood and beyond. Turin, Italy: Edizioni Minerva Medica; 2016. p. 43-56.

11. Waters CS, Annear B, Flockhart G, Jones I, Simmonds JR, Smith S, et al. Acceptance and Commitment Therapy for perinatal mood and anxiety disorders: a feasibility and proof of concept study. *British Journal of Clinical Psychology*. 2020;59:461-479.

12. Monteiro F, Fonseca A, Pereira M, Alves S, Canavarro MC. What protects at-risk postpartum women from developing depressive and anxiety symptoms? The role of acceptance-focused processes and self-compassion. *Journal of Affective Disorders*. 2019;246:522-529.

13. Bonacquisiti A, Cohen M, Schiller CE. Acceptance and commitment therapy for perinatal mood and anxiety disorders: development of an inpatient group intervention. *Archives of Womens Mental Health*. 2017;20:645-654.

14. Burns E, Schmied V, Fenwick J, Sheehan A. Liquid gold from the milk bar: constructions of breastmilk and breastfeeding women in the language and practices of midwives. *Social Science and Medicine*. 2012;75:1737-1745.
