# Supplementary material for: A brief gestalt intervention changes ultrasound measures of tongue movement during breastfeeding: case series
Source: BMC Pregnancy Childbirth. 2022 Feb 1;22:94. doi: 10.1186/s12884-021-04363-7 (PMC8808964; doi:10.1186/s12884-021-04363-7)
Supplement: Supplementary file 2 — Additional file 2. Fit and hold observations before and after a brief gestalt intervention, illustrated in photographs and videos. Description of key observations in photographs and videos before and after a brief gestalt intervention [file 12884_2021_4363_MOESM2_ESM.docx]

**Additional File 2**

**Fit and hold observations before and after a brief gestalt intervention, illustrated in photographs and videos**

*The fine plastic tube in some of the image and videos is attached to a manometer, which measures intra-oral vacuum. Vacuum measurement was not able to be consistently obtained in our samples and is therefore not included in the results.*

**Case A Fit and hold observations pre- and post-intervention**

Additional File 1: Case A Pre-intervention video *A-pre.mov*

- The mother sits upright (not in a semi-reclined or deckchair position).
- The mother’s right hand and arm hold the infant in the crook of her right arm so that the infant’s head and face rest against her upper arm (limiting the mother’s control over face-breast bury and micro-movements).
- The infant’s lumbar spine and pelvis are laterally rotated relative to cervical spine (so that the infant’s chest and abdomen are rotated away from mother’s body, not secured in close against the mother).
- The infant’s upper and lower lips are visible (not buried and unable to be seen due to a deep symmetrical face-breast bury).
- The infant dials up and pulls off the breast (indicating positional instability).
- When the infant pulls off the breast, the nipple with its shield drops lower relative to the infant’s mouth (suggesting there had been breast tissue drag when suckling).

Figure 4 Case A Post-intervention Photo 1 *A_post (1) JPEG*

- The infant’s head rests on the mother’s forearm with a gap between the baby’s forehead and the mother’s upper arm.
- A deep and symmetrical face-breast bury is visible.
- No gap is visible between nose and breast surface, and lips aren’t visible.

Figure 5 Case A Post-intervention Photo 2 *A_post (2) JPEG*

- The mother is semi-reclined.
- The infant’s head rests on the mother’s forearm, and the forehead doesn’t touch the mother’s upper arm.
- The infant’s abdomen, pelvis, and hips are wrapped around mother’s ribcage under her right breast.
- The infant’s spine is aligned, from skull and cervical spine to sacrum.
- The infant’s right arm is wrapped around the mother’s left side, allowing optimal face-breast bury and micro-movement control.

*Please note: in Figure 5, the woman requires firm support under her left elbow (e.g. small buckwheat pillow, pile of folded towels) as part of comprehensive (c.f. brief) gestalt intervention to protect her from neck and shoulder pain and to sustain infant positional stability.*

**Case C Fit and hold observations pre-intervention**

Figure 6 Case C Pre-intervention photo 1 *C_pre (1) JPEG*

- The infant’s lips are visible (indicating suboptimal face-breast bury, which compromises intra-oral breast tissue volume).
- The mother’s hand supports the infant’s neck and base of skull (which compromises the infant’s motoric postural control and reflexes).

Figure 7 Case C Pre-intervention photo 2 *C_pre (2) JPEG*

- The mother sits upright (not in a semi-reclined or deckchair position).
- The mother’s hand supports the infant’s neck and base of skull (which compromises the infant’s motoric postural control and reflexes).
- The infant’s lumbar spine and pelvis are laterally rotated relative to cervical spine (so that the infant’s chest and abdomen are rotated away from mother’s body, not secured in close against the mother).
- The infant’s lips are visible (indicating suboptimal face-breast bury, which compromises intra-oral breast tissue volume).

**Case D Fit and hold observations pre- and post-intervention**

*The mother was initially unable to bring the on to the breast, consistent with history of difficulty latching. An initial very brief intervention was eventually applied, in order to support infant coming onto the breast, prior to pre-intervention photos and videos. Pre-intervention images were taken after the initial very brief intervention, and prior to the standard brief gestalt intervention.*

Figure 8 Case D Pre-intervention Photo 1 *D_pre (1) JPEG*

- The mother’s hand holds the infant’s neck and base of skull (which compromises infant’s motoric postural control and reflexes).
- The infant’s lips are visible (indicating suboptimal face-breast bury, which compromises intra-oral breast tissue volume).

Figure 9 Case D Pre-intervention Photo 2 *D_pre (2) JPEG*

- The mother sits upright with slight forward lean (not in a semi-reclined or deckchair position).
- The mother’s hand holds the infant’s neck and base of skull (which compromises infant’s motoric postural control and reflexes).
- The infant’s lips are visible (indicating suboptimal face-breast bury, which compromises intra-oral breast tissue volume).
- A gap is visible between nose and breast (indicating suboptimal face-breast bury, which compromises intra-oral breast tissue volume).
- The infant’s lumbar spine and pelvis are laterally rotated relative to cervical spine (so that the infant’s chest and abdomen are rotated away from mother’s body, not secured in close against the mother).

Additional File 2 Case D Pre-intervention Video *D_pre.MP4*

- The mother may be trying to aim “nipple to nose” at the beginning (which does not benefit biomechanically and disrupts bringing the infant on).
- The mother’s hand supports the infant’s neck and base of skull (which compromises infant’s motoric postural control and reflexes).
- The infant’s lips are visible (indicating suboptimal face-breast bury, which compromises intra-oral breast tissue volume).
- A substantial gap is visible between nose and breast (indicating suboptimal face-breast bury, which compromises intra-oral breast tissue volume).
- The nipple moves laterally and upwards when infant comes off breast (suggesting there had been breast tissue drag when suckling).

Figure 10 Case D Post-intervention Photo 1 *D_post (1) JPEG*

- A deep face-breast bury is visible, with the lips not visible (allowing optimal intra-oral breast tissue volume).

Figure 11 Case D Post-intervention Photo 2 *D_post (2) JPEG*

- Deep face-breast bury and lips not visible (allowing optimal intra-oral breast tissue volume).
- Infant’s abdomen and hips wrapped around mother’s ribcage under her contralateral breast (older, longer babies may be more diagonal across a woman’s abdomen but chest and abdomen remain apposed to maternal torso).

Additional File 3 Case D Post-intervention Video *D_post.MOV*

- The mother is semi-reclined.
- The mother does not support the back of the infant’s head.
- There is a gap between the infant’s forehead and the mother’s upper arm.
- A deep and symmetrical face-breast bury is visible from a lateral view. (Some asymmetry of face-breast contact is still evident in the first part of this video, but subtle micro-movement support of mother’s forearm improves symmetry in second part of video – consent to touch was previously obtained).
- The infant’s nose rests on the breast surface but the infant is breathing comfortably.
- The infant is dialled down.

**Case E Fit and hold observations pre- and post-intervention**

Figure 12 Case E Pre-intervention Photo 1 E_pre (1) JPEG

- The mother sits upright or leans forward slightly (not in a semi-reclined or deckchair position).
- The mother’s hand supports the infant’s neck and base of skull (which compromises infant’s motoric postural control and reflexes).
- The infant’s thoracic and lumbar spine are extended (instead of flexed into a ribcage wrap, which optimises cervical extension and the biomechanics of suckling).
- The mother uses her hand to press her breast towards her midline and applies pressure on nipple shield to achieve “nipple to nose”.

Additional File 4 Case E Pre-intervention Video E_pre.MOV

- The mother sits upright or leans forward slightly (not in a semi-reclined or deckchair position).
- The mother’s hand supports the infant’s neck and base of skull (which compromises infant’s postural control and reflexes).
- The baby is dialled up.
- Mother applies some pressure on infant’s lower arm in order to support the breast and also initially uses her thumb on the breast to support the nipple with its shield, so that the breast and nipple sit higher than the natural breast fall (which results in breast tissue drag).
- The infant’s lips are visible.
- There is a gap between the infant’s nose and the breast.
- The breast falls lower than infant’s mouth when the infant pulls off (which suggests there was breast tissue drag when suckling).

*This infant is dialled up at the breast (note history of conditioned sympathetic nervous system hyperarousal or ‘conditioned dialling up’ at the breast). Sonographer gently restrains infant’s upper arm, which may contribute to dialling up.*

Figure 13 Case E Post-intervention Photo 1 *E_post (1) JPEG*

- A deep face-breast bury is visible.
- The infant’s lips are not visible.
- The infant’s nose rests lightly on the surface of breast.

Figure 14 Case E Post-intervention Photo 2 *E_post (2) JPEG*

- The mother is semi-reclined.
- The infant is in a tight ribcage wrap under the mother’s contralateral breast, so that the infant rests above her abdomen (which optimises biomechanics of suck and intra-oral breast tissue volume).
- The infant’s spine is aligned from the skull, through the cervical to the sacral spine, with some slight flexion of thoracic and lumbar spine and slight cervical extension (which optimise biomechanics of suck and intra-oral breast tissue volume).
- The infant’s head is supported by the mother’s forearm, which allows continuous micro-movements in response to her breast sensation and infant cues.
- The mother’s ipsilateral shoulder is relaxed, due to the use of a firm pillow to support her elbow. (Support by a cushion or pile of folded towels need to be very firm.)

Figure 15 Case A Post-intervention Photo 3 *E_post (3) JPEG*

- The infant has adequate symmetry of face-breast bury (although some asymmetry of cheek placement against the breast persists).
- The infant’s head rests on the mother’s forearm so that there is a gap between the infant’s forehead and her upper arm.
- The mother’s elbow rests on a firm pillow which does not interfere with the rest of her arm or the infant, but which gives her shoulder support.
